# 295 Multidisciplinary Reviews of Colectomy Surgeries Improve Accuracy of Surgical Site Infection Event Detection

**DOI:** 10.1017/ash.2026.10653

**Published:** 2026-06-23

**Authors:** Woojung Kim, Mimi Lee, Bomin Jeon, Pyoeng Gyun Choe, Sun Ju Chang

**Affiliations:** 1 Seoul National University Hospital; 2 Seoul National University Hospital, Seoul, Republic of Korea; 3 College of Nursing and Research Institute of Nursing Science, Seoul National University; 4 Seoul National University

## Abstract

**Background:** Carbapenem-resistant Acinetobacter baumannii (CRAB) is a critical multidrug-resistant pathogen. The Emergency Intensive Care Unit (EICU) is particularly vulnerable to transmission due to the high-pressure environment and need for immediate clinical decisions, often made with incomplete microbiological data. While patient-related factors are traditionally prioritized, the relative contribution of invasive procedures and environmental colonization pressure in this specialized setting remains under-quantified. This study aimed to identify the determinants of CRAB acquisition through a comprehensive, hierarchical analysis of patient, treatment, and environmental factors. **Methods:** A retrospective case–control study was conducted at a tertiary hospital from April 2020 to December 2023. Monthly indicators of CRAB acquisition were examined in relation to environmental characteristics using Spearman correlation. A four-block multivariable hierarchical logistic regression analysis, consisting of patient demographics, clinical characteristics, treatment factors, and environmental characteristics, was performed (48 cases, 144 controls) to identify independent risk factors for CRAB acquisition and to evaluate the model’s explanatory power. **Result:** During the study period, the monthly CRAB acquisition rate showed a median of 1.69 (range, 0.00–14.59) per 1,000 patient-days. This rate correlated positively with colonization pressure (daily mean number of CRAB-positive inpatients; r = .399, p = .007). Although hand hygiene compliance was high (median 93.8%; range 70.0–100.0%), it showed no correlation with CRAB acquisition rate (p = .753). In the hierarchical analysis, the model’s explanatory power improved significantly as treatment and environmental blocks were added (Nagelkerke R from .043 to .609). In the final model, carbapenem use was the strongest independent predictor (OR 8.58; 95% CI, 2.62–28.09; p p = .004), enteral nutrition (OR 6.36; p = .002), and mechanical ventilation (OR 4.79; p = .037). Colonization pressure also remained a significant independent risk factor (OR 3.56; p **Conclusion:** CRAB acquisition in the EICU is primarily driven by carbapenem-associated selection pressure, invasive procedures involving fluid exposure, and colonization pressure. Notably, the lack of correlation between high hand hygiene compliance and acquisition rates suggests that individual-level efforts alone are insufficient to mitigate risk in high-pressure environments. Consequently, effective prevention requires a paradigm shift from individual compliance toward organizational strategies. This includes strengthening treatment-related infection prevention bundles for high-risk invasive procedures and implementing dynamic environmental controls based on real-time colonization pressure.

